# Flavonoids from sour jujube leaves: Ultrasound-assisted extraction, UPLC-QQQ-MS/MS quantification, and ameliorative effect on DSS-induced ulcerative colitis in mice

**DOI:** 10.1016/j.ultsonch.2025.107279

**Published:** 2025-02-17

**Authors:** Hongke Wei, Xiaomin Wang, Jiaqi Wang, Shijie Ren, Luis A.J. Mur, Dong Lu, Duo Cao

**Affiliations:** a Shaanxi Key Laboratory of Research and Utilization of Resource Plants on the Loess Plateau, College of Life Sciences, Yan’an University, Yan’an 716000 Shaanxi, China; b Department of Life Sciences, Aberystwyth University, Ceredigion SY23 3DA, UK; c Shanghai Frontiers Science Center of TCM Chemical Biology, Institute of Interdisciplinary Integrative Medicine Research, Shanghai University of Traditional Chinese Medicine, Shanghai 201203, China

**Keywords:** Sour jujube leaves, Flavonoids, Ultrasound-assisted extraction, UPLC-QQQ-MS/MS, Ulcerative colitis

## Abstract

To valorize sour jujube (*Ziziphus acidojujuba*) leaves, this work focused on the extraction, quantification, and bioactivity assessment of flavonoids. First, ultrasound-assisted extraction (UAE) was employed to extract total flavonoids from sour jujube leaves (SJL-TF), with the procedure optimized through single-factor design and response surface methodology (RSM). SJL-TF yield reached 48.47 ± 0.36 mg/g under optimal circumstances, which included 61 % ethanol as extraction medium, an ultrasound power of 300 W, an extraction time of 33 min, and a liquid–solid ratio of 16 mL/g, showing higher extraction efficiency in comparison to the traditional Soxhlet extraction method. Scanning electron microscopy (SEM) analysis indicated that ultrasound treatment severely damaged the structural integrity of sour jujube leaves, which was more conducive to improving the SJL-TF yield. Then, polyamide resin chromatography was used to purify the crude SJL-TF extracts, increasing the SJL-TF purity by 3.2-fold to 74.58 ± 0.63 %. By developing and validating a UPLC-QQQ-MS/MS method, ten main flavonoids in SJL-TF extracts, including catechin, rutin, isoquercetin, narirutin, nicotiflorin, quercitrin, phlorizin, luteolin, quercetin, and apigenin, were successfully detected concurrently for quality control purposes. Furthermore, the purified SJL-TF showed a strong ameliorative effect on dextran sulfate sodium (DSS)-induced ulcerative colitis (UC) in mice, as evidenced by significant mitigation of colonic inflammation and pathological damage. Thus, this work will contribute to improving the application of sour jujube leaves, especially in the pharmaceutical sector.

## 1 Introduction

Sour jujube (*Ziziphus acidojujuba*) is a perennial shrub in the Rhamnaceae family, commonly found in temperate and subtropical regions of the northern hemisphere, especially in Northern China [1]. Its fruits can be enjoyed fresh or dried and serve as food additives to enhance flavor. Additionally, its dried ripe seeds, known as “Spine date seeds”, have been utilized in traditional Chinese medicine for thousands of years to promote sleep, nourish the liver, calm the mind, soothe the nerves, and reduce sweating [2]. Conversely, sour jujube leaves have garnered less attention and are often regarded merely as agricultural byproducts. Recently, a portion of sour jujube leaves has been transformed into functional beverages, gaining popularity among consumers [3]. Sour jujube leaves, known for their high flavonoid content, are recognized as a valuable natural source of these beneficial compounds [4], [5]. Thus, the exploration of sour jujube leaves, particularly their flavonoids, which have yet to be fully explored in agricultural practices, is well-aligned with the principles of the circular economy and sustainable development.

Traditional extraction methods for flavonoids, such as decoction, maceration, heat reflux, and Soxhlet extraction, often require considerable time or organic solvents, resulting in potential thermal degradation and low yields [6]. As an alternative, ultrasound-assisted extraction (UAE) is increasingly being recognized as a promising green extraction technology, gaining more attention [7]. Ultrasound generates cavitation, vibration, crushing, and mixing effects, which can effectively disrupt plant cell walls. This facilitates the release of target compounds and greatly enhances the mass transfer process [8]. This innovative technique not only reduces the risk of heat-induced degradation of sensitive bioactive ingredients but also creates a more controlled extraction environment. Consequently, UAE preserves the efficacy of the extracted compounds while enhancing the quality of the final products [9]. Thus, due to high extraction yield, low solvent usage, eco-friendliness, and short extraction duration, UAE has become widely utilized for extracting flavonoids from plant materials, such as *Pteris cretica* L. [6], *Astragalus membranaceus* stems and leaves [10], peanut shells [11], and pomelo peels [12]. In addition, selecting appropriate UAE parameters, such as extraction time, temperature, liquid–solid ratio, ultrasound power, particle size, and the number of extraction cycles, is crucial for achieving maximum extraction yield. Recently, response surface methodology (RSM), a multivariate statistical method that fits a polynomial model to experimental data, has been widely used to optimize the process parameters of UAE for flavonoids [6], [10], [12].

Ultra performance liquid chromatography (UPLC) improves upon LC by utilizing columns with smaller particle sizes and operating at higher pressures, resulting in faster separations and enhanced resolution [13]. In quantitative analysis, the triple quadrupole (QQQ) mass spectrometer is widely recognized as a highly efficient and primary tool [14]. Higher collision frequency significantly boosts the conversion of precursor ions to product ions, enhancing sensitivity, which allows for the detection of low-abundance compounds in complex matrices [15]. The versatility of the QQQ mass spectrometer, which enables smooth transitions among operational modes such as single ion monitoring (SIM), selective reaction monitoring (SRM), and multiple reaction monitoring (MRM), underscores its efficacy across a wide range of analytical applications [16]. To guarantee the accurate detection of multiple analytes, the MRM mode is highly preferred, as it depends on predetermined transitions from precursor ions to product ions. UPLC-QQQ-MS/MS operating in MRM mode has emerged as a popular method for quantifying phytochemicals [17], [18].

Ulcerative colitis (UC), is a chronic condition marked by persistent immune-mediated inflammation of the gastrointestinal tract that disrupts gut homeostasis and manifests in symptoms such as abdominal pain, diarrhea, and weight loss [19]. The pathogenesis of UC is multifactorial, involving a complex interplay of immune system dysregulation, environmental triggers, and genetic predisposition [20]. Traditional pharmacological treatments for UC, primarily involving medications like sulfasalazine and mesalamine, can effectively alleviate symptoms, but they often come with side effects that may seriously affect the quality of life [21]. In light of these challenges, there is growing interest in alternative therapeutic approaches, particularly the use of natural products. Recent studies indicate that flavonoids can effectively manage UC through modulating immune responses, disrupting inflammatory pathways, regulating cytokines, balancing gut microbiota, and protecting the intestinal mucosal barrier [22]. These promising findings greatly highlight the potential of developing flavonoid compounds into effective and safer medications UC.

To investigate the potential of sour jujube leaves as a renewable source of bioactive compounds, the UAE technique was employed to extract total flavonoids from sour jujube leaves (SJL-TF), with the process optimized using RSM. Afterwards, a UPLC-QQQ-MS/MS method was established to simultaneously quantify ten major flavonoid compounds. Finally, the ameliorative effects of purified total flavonoids on UC were evaluated in a mouse model of DSS-induced colonic inflammation. This research will undoubtedly enhance the comprehensive utilization of sour jujube resources.

## 2 Materials and methods

### 2.1 Chemicals and reagents

All standards were obtained from Aladdin Co., Ltd. (Shanghai, China). Polyamide resin (60–100 mesh) was sourced from Nantong Hairuo Chemical Co., Ltd. (Nantong, China). Acetonitrile, formic acid (LC-MS grade), and hematoxylin & eosin (H&E) staining kit were obtained from Sigma-Aldrich (Shanghai, China). DSS was purchased from MP Biomedicals, Inc. (California, USA). Myeloperoxidase (MPO), TNF-α, IL-6, IL-10, and IL-12 ELISA kits were obtained from Thermo Fisher Scientific Inc. (Shanghai, China).

### 2.2 Plant material

Fresh sour jujube leaves were manually harvested in May 2023 from Yan’an City, Shaanxi Province, China. The leaves were dried at room temperature, ground, passed through a 40-mesh sieve, and then kept in a desiccator at 4 °C until further analysis.

### 2.3 Ultrasound-assisted extraction of SJL-TF

#### 2.3.1 Extraction procedure

Ultrasound generator (KQ-400DE, 40 kHz) was utilized for the UAE of SJL-TF. A total of 2.0 g of powdered sour jujube leaves were placed in a conical flask and subjected to extraction using a specified solvent, ultrasound power, duration, and liquid–solid ratio, across three extraction cycles. Upon completion of the extraction, the extract solutions were combined, centrifuged at 10,000 rpm for 5 min, and stored at 4 °C until further analysis.

#### 2.3.2 Single-factor design

Based on the findings from the literature survey and preliminary experiments, four key factors influencing the yield of SJL-TF were identified for investigation, including ethanol concentration, ultrasound power, extraction time, and liquid-to-solid ratio. Specifically, a single-factor design was conducted to examine the effects of varying ethanol concentration (30 %–90 %), ultrasound power (160–400 W), extraction time (10–40 min). and liquid–solid ratio (10–30 mL/g) on the yield of SJL-TF, which aimed to provide a comprehensive insight into how each factor contributes to optimizing the extraction process.

#### 2.3.3 Design and analysis of response surface experiments

Based on the insights gained from the single-factor design, the range of variables was refined. Subsequently, a response surface experimental design was implemented, focusing on the interactions among four key independent variables. To optimize the extraction process and enhance the yield and efficiency in the production of SJL-TF, a Box-Behnken design (BBD) was employed. Table 1 presents the parameters and outcomes of 29 randomized experiments, and the data from the BBD were then analyzed using the following polynomial model (1). To confirm the adequacy of the predictive model, *p*-values for the model and lack-of-fit, as well as the correlation coefficient (*R*^2^), adjusted *R*^2^, and coefficient of variation (C.V.), were analyzed using analysis of variance (ANOVA). Process optimization was performed through a desirability function. Finally, three parallel validation tests under the established optimal conditions were executed.

(1) $$Y=\beta_{\text{0}}+\sum_{i=1}^{k}\beta_{i}X_{i}+\sum_{i=1}^{k}\beta_{\mathrm{ii}}X_{i}^{\text{2}}+\sum_{i=1}^{k}\sum_{j=i+1}^{k-1}\beta_{\mathrm{ij}}X_{i}X_{j}$$

Where, *β*_0_ indicates the intercept, while *β*_i_, *β*_ii_, and *β*_ij_ represent the linear, quadratic, and interaction effects, respectively. The independent variables are denoted by *X*_i_ and *X*_j_, with *k* representing the total number of independent variables involved.

**Table 1:** BBD experiments and results.

Run	*X*_1_ (%)	*X*_2_ (W)	*X*_3_ (min)	*X*_4_ (mL/g)	*Y* (mg/g)
1	70	280	30	20	37.53
2	70	320	30	15	41.34
3	50	360	30	20	28.04
4	60	280	35	20	42.31
5	60	320	25	25	40.86
6	70	360	30	20	33.87
7	60	320	30	20	45.12
8	60	280	30	15	43.21
9	50	320	30	25	39.78
10	60	360	30	15	30.75
11	50	320	35	20	41.53
12	60	320	30	20	45.21
13	60	320	25	15	32.46
14	60	320	30	20	47.29
15	50	320	30	15	34.75
16	50	320	25	20	29.07
17	60	360	35	20	31.15
18	60	360	25	20	28.59
19	60	280	30	25	42.61
20	70	320	25	20	29.63
21	60	280	25	20	31.72
22	50	280	30	20	37.08
23	60	320	35	25	38.42
24	60	360	30	25	38.75
25	70	320	30	25	40.74
26	70	320	35	20	42.03
27	60	320	35	15	45.09
28	60	320	30	20	46.05
29	60	320	30	20	47.34

*Notes*: *X*_1_ – EtOH concentration; *X*_2_ – ultrasound power; *X*_3_ – extraction time; *X*_4_ – liquid–solid ratio *Y* – yield.

### 2.4 Conventional Soxhlet extraction

Powdered sour jujube leaves (2.0 g) were placed in a Soxhlet extraction apparatus containing 32 ml of 61 % ethanol, with extraction conducted for 2 h per cycle and repeated three times. After the extraction was completed, the extract solutions were pooled and centrifuged at 10,000 rpm for 5 min, and then stored at 4 °C for further analysis.

### 2.5 Scanning electron microscopy (SEM) analysis

Subsequent to the extraction process, the sour jujube leaves were meticulously rinsed with ultrapure water and subjected to freeze-drying prior to being coated with gold. The structural morphology of the leaves was then examined using SEM. High-resolution images were captured from randomly selected regions of each sample, with the dimensions of these regions meticulously chosen to adequately represent the overall characteristics of the sample.

### 2.6 Enrichment of SJL-TF by polyamide resin chromatography

The purification of SJL-TF was conducted using a glass column packed with polyamide resin, featuring an inner diameter of 20 mm and a height-to-diameter ratio of 1:7. The sample solution was prepared by suspending SJL-TF extract in H_2_O, maintaining a concentration of 1.0 mg/mL. For the leakage curve study, feed rates of 2, 3, and 4 BV/h were examined, with effluent collected simultaneously during the analysis of SJL-TF concentration. For the elution curve study, a flow rate of 3 BV/h was used, with the elution program consisting of 5 BV of H_2_O, followed by 5 BV each of 15 %, 45 %, and 75 % ethanol, and 8 BV of 95 % ethanol. Effluent was collected concurrently to monitor SJL-TF concentration.

### 2.7 Determination of total flavonoids content

The determination of total flavonoids content was conducted based on a previous report [12]. A 100 μL aliquot of the sample was mixed with 30 μL of 5 % NaNO_2_ solution. This mixture was allowed to react for 6 min before the addition of 30 μL of 10 % AlCl_3_ solution. After another 6 min, 200 μL of NaOH solution and 640 uL of H_2_O were added. The resulting solution was mixed and then left to stand at room temperature for 20 min. Then, absorbance of a 250 μL sample from this reaction, dispensed into a 96-well plate, was measured at 510 nm using a SpectraMax® iD5 microplate reader. A quercitrin calibration curve was constructed, yielding a linear equation of *y* = 0.0010*x* + 0.0283 (*R*^2^ = 0.9997), where *y* represents the absorbance, and *x* represents the concentration, across a concentration range of 100–500 μg/mL.

### 2.8 UPLC-QQQ-MS/MS quantitation

#### 2.8.1 Analysis conditions

UPLC analysis was conducted using an Agilent 1260 Infinity II LC system equipped with an ACQUITY UPLC BEH C18 (2.1 × 100 mm, 1.7 μm). The sample was injected at a volume of 1.0 μL, with the column temperature kept at 35°C. The mobile phase comprised 0.2 % formic acid in H_2_O (phase A) and acetonitrile (phase B), delivered at 0.4 mL/min using the following elution profile: 0–7 min, 5–15 % B; 7–15 min, 15–28 % B; 15–17 min, 28–100 % B; 17–18 min, 100–5 % B; 18–20 min, 5 % B. Mass spectrometry data acquisition was carried out using an Agilent G6470B triple-quadrupole mass spectrometer with a negative ESI source. The MRM parameters for the target analytes in negative ionization mode were optimized using Agilent MassHunter Optimizer and are detailed in Table 2.

**Table 2:** MRM parameters for quantification of ten flavonoid compounds.

Compounds	Formula	Retention time (min)	Precursor Ion (*m*/*z*)	Product Ion (*m*/*z*)	Fragmentor (V)	Collision Energy (eV)
Catechin	C_15_H_14_O_6_	4.812	289.07	119	245.1	13
Rutin	C_27_H_30_O_16_	9.814	609.14	238	300.1	41
Isoquercetin	C_21_H_20_O_12_	10.193	463.09	228	300.1	29
Narirutin	C_27_H_32_O_14_	10.792	579.17	228	271.1	25
Nicotiflorin	C_27_H_30_O_15_	11.061	593.15	233	285.1	33
Quercitrin	C_21_H_20_O_11_	11.594	447.09	228	300.1	25
Phlorizin	C_21_H_24_O_10_	12.535	435.13	176	273.1	13
Luteolin	C_15_H_10_O_6_	15.349	285.04	171	133	37
Quercetin	C_15_H_10_O_7_	15.501	301.03	176	151	25
Apigenin	C_15_H_10_O_5_	16.484	269.04	176	117.1	41

#### 2.8.2 Method validation

Method validation was performed based on our previous study [23], encompassing various methodological parameters such as limit of quantification (LOQ), limit of detection (LOD), precision, and recovery.

### 2.9 Ameliorative effect on DSS-induced UC in mice

#### 2.9.1 Experimental modeling and drug intervention

In the present study, six-week-old male C57BL/6 mice were utilized as the experimental model. After an acclimatization period of 7 days to minimize stress and allow for adaptation to the laboratory environment, the mice were randomly assigned to one of six groups (*n* = 5), including control group, model group, mesalazine group, SJL-TF-L group, SJL-TF-M group, and SJL-TF-H group. The control group received water, serving as a baseline for comparison, while the other groups were given a 2.5 % DSS solution in water for 6 straight days. From day 1 to day 9, mice in the treatment groups received oral gavage with specific dosages: the mesalazine group received 500 mg/kg/day of mesalazine granules, while the SJL-TF-L, SJL-TF-M, and SJL-TF-H groups received SJL-TF at doses of 50, 100, and 200 mg/kg/day, respectively. This regimen was based on preliminary studies showing effective concentrations for alleviating UC symptoms. At the end of the treatment period, all mice fasted overnight. On day 10, the mice were anesthetized and euthanized by cervical dislocation to collect tissues for histological and biochemical analysis.

#### 2.9.2 Related treatment effect detection and analysis

The disease activity index (DAI) was calculated daily by combining scores from assessments of body weight, stool consistency, and colonic hemorrhage, based on previously defined scoring criteria [24]. Furthermore, colon length and spleen weight were measured. Myeloperoxidase (MPO) activity, and the levels of pro-inflammatory cytokines (TNF-α, IL-6, and IL-12) and anti-inflammatory cytokine (IL-10) in colon tissues were assessed using corresponding ELISA kits. Colon tissues were fixed in 4 % paraformaldehyde, dehydrated using an ethanol gradient, embedded in paraffin, and stained with hematoxylin and eosin (H&E) for microscopic analysis of the colon architecture. The histological score was referenced from a previous report [25].

### 2.10 Statistical analysis

Data are presented as mean ± standard deviation from at least three replicates. Design-Expert software was used for the response surface design and analysis. Statistical analysis employed one-way or two-way ANOVA, with significance set at *p* < 0.05.

## 3 Results and discussion

### 3.1 Analysis of single-factor experiment

#### 3.1.1 Ethanol concentration

**Fig. 1:**
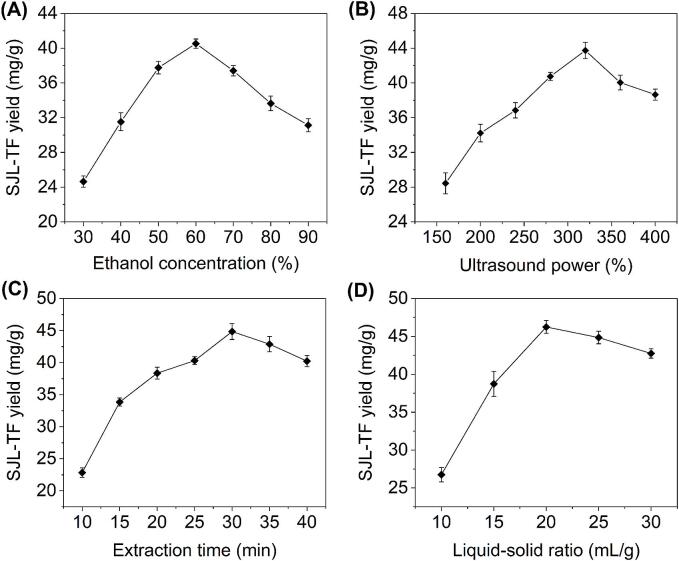
Effects of various parameters on SJL-TF yield. (A) ethanol concentration; (B) ultrasound power; (C) extrcation time; (D) liquid–solid ratio.

Ethanol aqueous solutions were commonly used as extraction solvents for flavonoid compounds, primarily due to their excellent solubility and strong cellular permeability. As illustrated in Fig. 1A, it was found that as the ethanol concentration initially increased, the SJL-TF yield exhibited an upward trend, peaking at 60 % (40.53 ± 0.52 mg/g). This increase was attributed to the enhanced solubility of flavonoids at higher ethanol concentrations, which simultaneously minimized the interference of impurities such as polysaccharides. However, when the ethanol concentration exceeded 60 %, a decline in the SJL-TF yield was observed, as higher ethanol concentrations also facilitated the leaching of less polar impurities, such as pigments [6]. A similar trend has been described in the UAE of flavonoids from *Astragalus membranaceus* stems and leaves [10] and *Daphne genkwa* [26]. Thus, 60 % ethanol emerged as the optimal concentration for extracting SJL-TF, in accordance with the principle of similar compatibility.

#### 3.1.2 Ultrasound power

Ultrasound power is a key parameter influencing extraction efficiency, directly tied to energy transfer and solvent-sample interaction. As shown in Fig. 1B, when the ultrasound power increased from 160 to 320 W, the yield of SJL-TF rose from 28.44 ± 1.22 to 43.74 ± 0.94 mg/g. However, further increases in ultrasound power led to a decline in SJL-TF yield. A comparable tendency has been observed in the UAE of flavonoids from olive leaves [27]. This phenomenon could be attributed to the initial phase where increased power enhanced the intensity of energy transfer, effectively disrupting cell walls and breaking down plant tissues, resulting in improved extraction efficiency. However, if the power was excessively high, it could result in the degradation of the target flavonoids, thereby reducing the extraction yield [28]. Therefore, the ideal ultrasound power was determined to be 320 W.

#### 3.1.3 Extraction time

Optimizing extraction time is crucial for enhancing yield while simultaneously reducing energy and time costs. As shown in Fig. 1C, increasing the extraction time from 10 to 30 min resulted in a rise in SJL-TF yield from 22.85 ± 0.74 to 44.84 ± 1.24 mg/g. Nevertheless, prolonging the extraction duration past this stage resulted in a decline in SJL-TF yield. Similar patterns have been observed in the UAE of flavonoids from *Pteris cretica* [6] and olive leaves [27]. This phenomenon occurs because an appropriate extraction time facilitates the effective release of target flavonoid compounds, while prolonged extraction can intensify the thermal effect, resulting in the thermal degradation of the flavonoid compounds and a subsequent decrease in SJL-TF yield [26]. Consequently, 30 min was deemed the optimal extraction time.

#### 3.1.4 Liquid-solid ratio

The selection of the liquid–solid ratio is crucial for both extraction yield and production cost. As presented in Fig. 1D, the SJL-TF yield was highly sensitive to the liquid–solid ratio within the range of 10–20 mL/g, showing an increasing trend from 26.75 ± 0.96 to 46.24 ± 0.85 mg/g. However, when the liquid–solid ratio exceeded 20 mL/g, the SJL-TF yield began to decline gradually. Similar results have been reported for the UAE of flavonoids from the stems and leaves of *Astragalus membranaceus* [10]. Generally, a larger liquid–solid ratio improves the dissolution of the target components due to the increased concentration gradient and cavitation effect, which enhances the diffusion rate and extraction yield [28], [29]. However, an excessively high liquid–solid ratio may lead to a decrease in ultrasonic density and increased sound wave attenuation, which is detrimental to the extraction of target components [30]. Thus, a liquid–solid ratio of 20 mL/g was determined to be optimal.

### 3.2 Response surface analysis and model validation

#### 3.2.1 Response surface analysis

Through binomial fitting analysis of the BBD experimental results, the following prediction model (2) was obtained, expressed in terms of coded factors. To further assess the variability and appropriateness of the new prediction model, an analysis of variance (ANOVA) was conducted. As indicated in Table 3, the model demonstrated statistical significance (*p* < 0.0001), while the “lack of fit” was found to be nonsignificant (*p* > 0.05), confirming its adequacy. Significant coefficients were observed for factors *X*_1_, *X*_2_, and *X*_3_, as well as interactions *X*_2_*X*_4_, *X*_3_*X*_4_, and the quadratic terms *X*_1_^2^, *X*_2_^2^, and *X*_3_^2^ (*p* < 0.05). The determination coefficient (*R*^2^) was calculated at 0.9528, suggesting that the regression model accounts for 95.28 % of the total variation in the data. The adjusted *R*^2^ value of 0.9055 further indicated a strong correlation between the experimental and predicted outcomes. Additionally, the coefficient of variation (C.V.%) was recorded at 4.91, reflecting satisfactory repeatability of the predictions generated by the model [31], [32].

(2) $$\begin{array}{c} Y=46.20+1.24X_{1}-3.61X_{2}+4.02X_{3}+1.13X_{4}+1.35X_{1}X_{2}-0.015X_{1}X_{3}-1.41X_{1}X_{4}\\ -2.01X_{2}X_{3}+2.15X_{2}X_{4}-3.77X_{3}X_{4}-5.40X_{1}^{2}-6.62X_{2}^{2}-5.71X_{3}^{2}-1.23X_{4}^{2} \end{array}$$

**Table 3:** ANOVA for quadratic model.

Source	Sum of Squares	df	Mean Square	*F*-value	*p*-value
Model	1000.29	14	71.45	20.17	< 0.0001
*X*_1_	18.48	1	18.48	5.21	0.0385
*X*_2_	156.31	1	156.31	44.12	< 0.0001
*X*_3_	193.60	1	193.60	54.64	< 0.0001
*X*_4_	15.32	1	15.32	4.32	0.0564
*X*_1_*X*_2_	7.24	1	7.24	2.04	0.1749
*X*_1_*X*_3_	0.0009	1	0.0009	0.0003	0.9875
*X*_1_*X*_4_	7.92	1	7.92	2.24	0.1570
*X*_2_*X*_3_	16.12	1	16.12	4.55	0.0511
*X*_2_*X*_4_	18.49	1	18.49	5.22	0.0385
*X*_3_*X*_4_	56.78	1	56.78	16.02	0.0013
*X*_1_^2^	189.04	1	189.04	53.35	< 0.0001
*X*_2_^2^	284.35	1	284.35	80.25	< 0.0001
*X*_3_^2^	211.84	1	211.84	59.79	< 0.0001
*X*_4_^2^	9.77	1	9.77	2.76	0.1190
Residual	49.60	14	3.54		
Lack of fit	44.95	10	4.49	3.86	0.1024
Pure error	4.66	4	1.16		
Cor total	1049.89	28			

*Notes*: *X*_1_ – EtOH concentration; *X*_2_ – ultrasound power; *X*_3_ – extraction time; *X*_4_ – liquid–solid ratio; df – degrees of freedom.

**Fig. 2:**
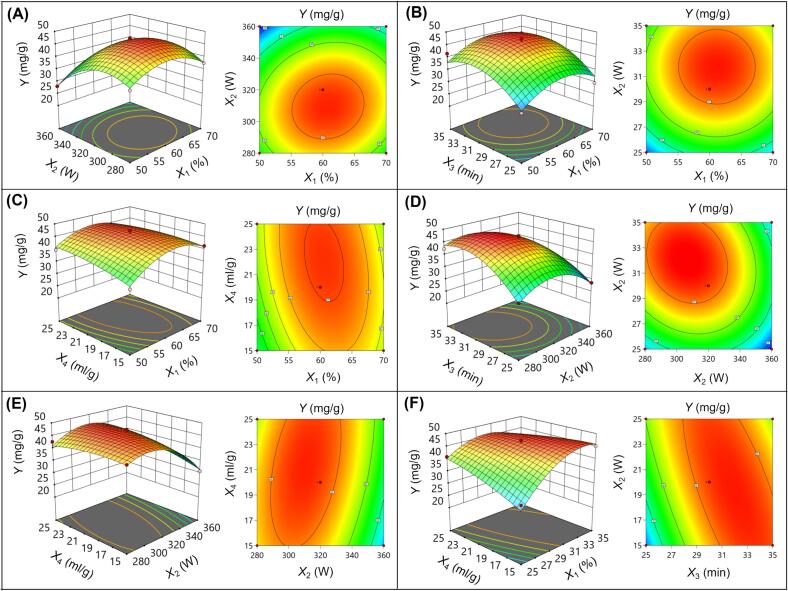
Response surface and contour plots depicting the interactions between the following pairs of variables: *X*_1_ and *X*_2_ (A), *X*_1_ and *X*_3_ (B), *X*_1_ and *X*_4_ (C), *X*_2_ and *X*_3_ (D), *X*_2_ and *X*_4_ (E), and *X*_3_ and *X*_4_ (F) on the SJL-TF yield.

Admittedly, response surfaces and contour plots offer a clear and intuitive visualization of the interactions between two factors while holding the other factor constant, typically at zero. Fig. 2A-F illustrates the interactions between the factors *X*_1_ and *X*_2_, *X*_1_ and *X*_3_, *X*_1_ and *X*_4_, *X*_2_ and *X*_3_, *X*_2_ and *X*_4_, and *X*_3_ and *X*_4_, respectively, on the SJL-TF yield. It was observed that the SJL-TF yield exhibited a trend of initially increasing and then decreasing with the rise of *X*_1_, *X*_2_, and *X*_3_ within the studied range, while no significant trend was evident with changes in *X*_4_, further corroborating the significant effects of *X*_1_, *X*_2_, and *X*_3_ on SJL-TF yield as indicated in Table 3, and highlighting the insignificance of the effect of *X*_4_. Additionally, the 3D surface plot of *X*_3_ and *X*_4_ appeared notably steep, and the contour plot was significantly non-circular. Furthermore, the interaction between *X*_2_ and *X*_4_ exhibited a similar trend, further confirming the significant impact of the interactions between *X*_2_ and *X*_4_, as well as *X*_3_ and X_4_, on SJL-TF yield, as indicated in Table 3 (*p* < 0.05).

#### 3.2.2 Model validation

The ideal combination of independent factors to obtain the predicted maximum yield of SJL-TF at 48.416 mg/g, as determined by the desirability function, included an ethanol concentration of 61.46 %, ultrasound power of 302.03 W, extraction time of 33.17 min, and a liquid–solid ratio of 16.26 mL/g. To further evaluate the reliability of the predictive model developed in this study, slight modifications were made to the extraction conditions for practical application. Specifically, the UAE of SJL-TF was conducted at an ethanol concentration of 61 %, ultrasound power of 300 W, extraction time of 33 min, and a liquid–solid ratio of 16 mL/g. This resulted in a yield of 48.47 ± 0.36 mg/g, which matched the predicted value closely, indicating that the prediction model was stable and reliable.

### 3.3 Comparative analysis with conventional Soxhlet extraction

**Fig. 3:**
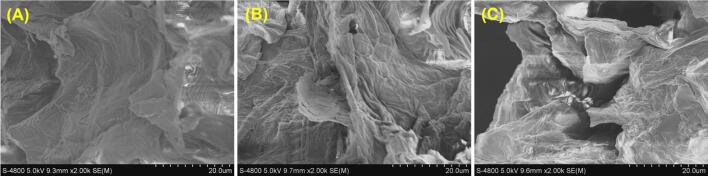
SEM images of sour jujube leaves before treatment (A), after Soxhlet extraction (B), and after ultrasound-assisted extraction (C).

The CFL-TP yield achieved through HRE was 35.79 ± 0.42 mg/g, which was markedly lower than that obtained via UAE. A comparable trend has been observed in the extraction of flavonoids from peanut shells [11] and *Millettia speciosa* Champ. [33]. Furthermore, SEM was used to observe the morphological changes in sour jujube leaves before and after applying various extraction methods. Fig. 3 revealed that untreated sour jujube leaves exhibited a relatively smooth surface texture, characterized by the absence of debris or pores, indicating that the leaves retain structural integrity and natural protective barriers. In contrast, significant alterations in the surface structure after extraction highlight the profound impact of extraction methods on leaf morphologies. The damage, which included the formation of debris and pores, disrupted cellular architecture and compromised cell wall integrity, thereby enhancing the release of target compounds during extraction. It was evident that the degree of tissue damage in the leaves after UAE was considerably more severe than that observed with the Soxhlet extraction method, which can be attributed to the use of high-frequency sound waves that generate collapsing cavitation bubbles, creating localized heat and pressure that disrupt plant tissue and facilitate the diffusion of flavonoid compounds [34].

### 3.4 Enrichment of SJL-TF

**Fig. 4:**
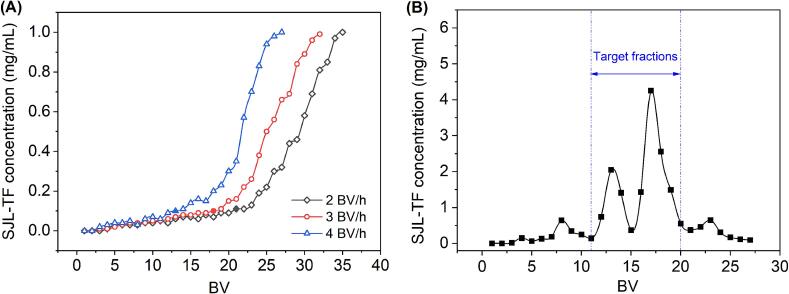
Leakage curves (A) and elution curves (B).

As shown in Fig. 4A, the loading capacities at 2, 3, and 4 BV/h reached their respective leakage points at 21, 18, and 13 BV. The decrease in loading capacity with increasing flow rates was attributed to the reduced contact time between the flavonoid compounds and the polyamide resin, leading to inadequate adsorption and early leakage [35]. When comparing the loading amounts at flow rates of 2 BV/h and 3 BV/h, the differences were found to be slight. Considering this observation along with the necessity for operational efficiency, 3 BV/h was determined to be the most appropriate for sample loading. Regarding elution, Fig. 4B illustrated that H_2_O was largely ineffective in eluting the flavonoids adsorbed on the polyamide resin, while 15 % ethanol managed to elute only a small fraction of the flavonoids, indicating that 5 BV of H_2_O followed by 5 BV of 15 % ethanol could effectively remove more polar impurities. In contrast, a 45 % ethanol effectively eluted the adsorbed flavonoid compounds, whereas a 75 % ethanol demonstrated the highest efficiency. Additionally, 95 % ethanol was able to elute a small residual amount of flavonoids. Consequently, the fractions collected from the 45 % and 75 % ethanol elutions, specifically those from 11 to 20 BV as shown in Fig. 4B, were collected, concentrated, and freeze-dried, resulting in a flavonoid-rich product with a purity of 74.58 ± 0.63 %, which was 3.2 times higher than the initial purity before purification, and a recovery of 78.13 ± 0.55 %. Results indicated the effectiveness of the polyamide resin chromatography developed in this study for purifying SJL-TF.

### 3.5 Quantitative analysis of ten main flavonoid compounds

#### 3.5.1 Verification of quantitative method

Table 4 shows that the established method provided excellent linear correlations (*R*^2^ > 0.99) for the ten flavonoids within the tested concentration range. LODs ranged from 0.95 to 15.28 ng/mL, and LOQs from 3.12 to 42.35 ng/mL. Intra-day and inter-day RSDs were ≤3.66 % and ≤4.84 %, respectively, with recovery between 96.93 % and 104.15 %. These results indicated the high sensitivity and precision of the quantitative method developed.

**Table 4:** Method validation for the quantification of ten flavonoid compounds and their contents.

Compounds	Regression equation	Linear range (µg/mL)	*R*^2^	LOD (ng/mL)	LOQ (ng/mL)	Precision (RSD, %)		Recovery (%)	Content (%)	
						Intra-day	Inter-day		BP	AP
Catechin	*y* = 16353*x*-5249	1–10	0.9991	15.28	42.35	2.74	4.57	98.11	2.42 ± 0.15	9.40 ± 0.14
Rutin	*y* = 39872*x*-15314	2–20	0.9992	2.12	6.40	1.51	2.94	101.75	2.88 ± 0.12	13.75 ± 0.36
Isoquercetin	*y* = 20578*x*-11957	0.5–5	0.9994	4.55	13.82	3.02	4.71	98.86	0.69 ± 0.04	2.14 ± 0.12
Narirutin	*y* = 41972*x*-4962	0.25–5	0.9986	1.92	5.66	2.69	4.30	96.93	0.33 ± 0.02	1.92 ± 0.03
Nicotiflorin	*y* = 38464*x*-8835	0.5–20	0.9989	4.82	14.33	2.12	4.84	99.96	1.27 ± 0.04	7.18 ± 0.51
Quercitrin	*y* = 10831*x*-6708	0.25–5	0.9990	6.60	22.14	1.79	2.67	100.35	0.81 ± 0.06	2.96 ± 0.03
Phlorizin	*y* = 38057*x*-6013	0.25–5	0.9993	10.35	34.92	2.34	3.50	104.15	0.91 ± 0.02	3.05 ± 0.26
Luteolin	*y* = 192684*x*-9815	0.02–2	0.9987	1.90	5.74	1.89	4.06	98.03	0.42 ± 0.04	1.13 ± 0.04
Quercetin	*y* = 25862*x* + 4742	0.01–1	0.9992	12.35	40.50	2.12	3.77	97.64	0.19 ± 0.03	0.82 ± 0.03
Apigenin	*y* = 165939*x*-16530	0.01–1	0.9988	0.95	3.12	3.66	3.95	98.70	0.15 ± 0.02	0.36 ± 0.01

*Notes*: LOD – limit of detection; LOQ – limit of quantification; RSD – relative standard deviation; BP – before purification; AP – after purification

#### 3.5.2 Quantification of ten flavonoids

**Fig. 5:**
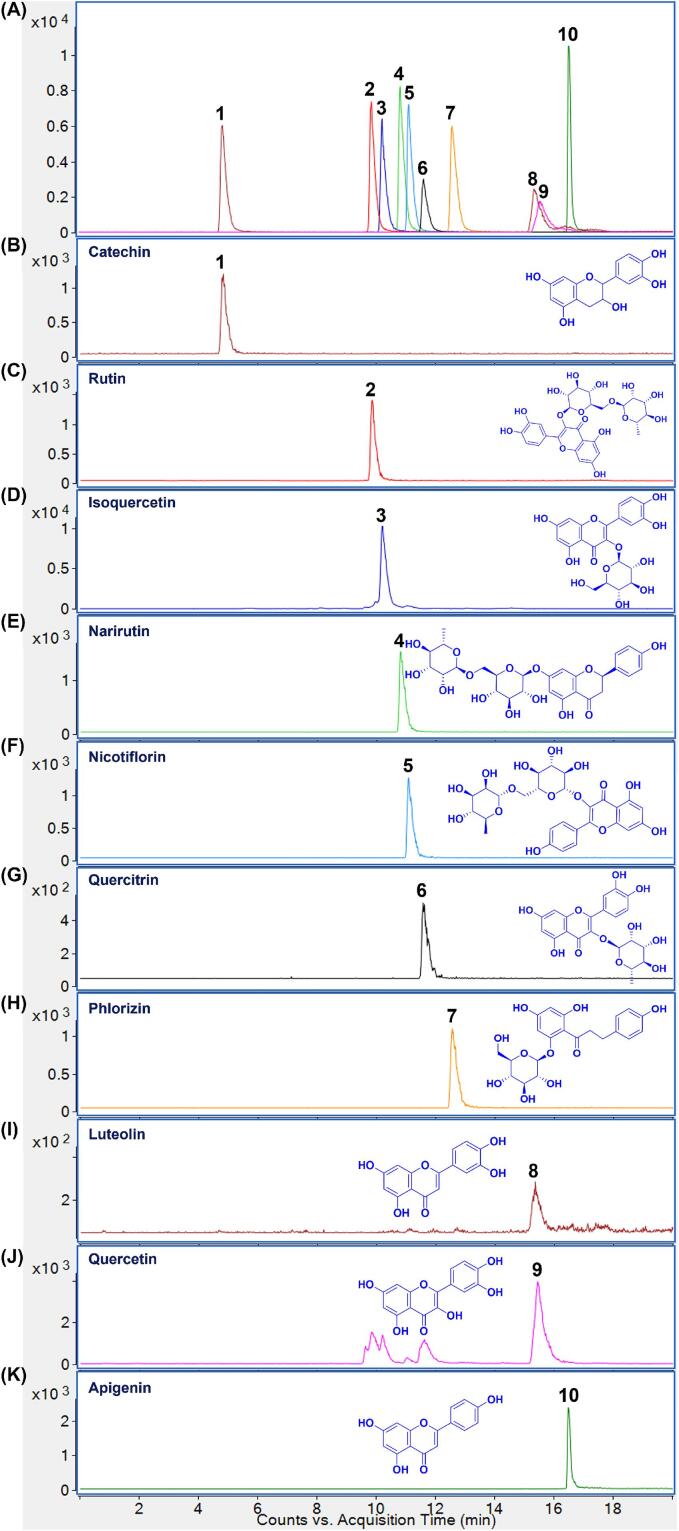
Chromatogram of extracted MRM transitions from a mixture of ten flavonoid standards (A) and representative chromatograms of extracted MRM transitions from sour jujube leaf extracts for the ten flavonoid compounds (B–K).

The merged MRM transitions for ten flavonoid standards (catechin, rutin, isoquercetin, narirutin, nicotiflorin, quercitrin, phlorizin, luteolin, quercetin, and apigenin) are displayed in Fig. 5A. Meanwhile, Fig. 5B–K present the representative MRM transition chromatograms of flavonoids in the SJL-TF extracts. As shown in Table 4, before purification, the SJL-TF extracts contained relatively high levels of rutin (2.88 ± 0.12 %), followed by catechin (2.42 ± 0.15 %) and nicotiflorin (1.27 ± 0.04 %). while, after purification, the concentrations increased significantly, with rutin at 13.75 ± 0.36 %, catechin at 9.40 ± 0.14 %, and nicotiflorin at 7.18 ± 0.51 %. After a single step of polyamide resin chromatography, the contents of ten analytes elevated by 2.40–5.82 times, with narirutin showing the highest increase (5.82-fold), followed by nicotiflorin (5.65-fold), rutin (4.77-fold) and quercetin (4.32-fold), raising the total content of these flavonoids from 10.07 % to 42.71 %. Therefore, it can be concluded that the developed polyamide column chromatography process was suitable for the purification of flavonoids from sour jujube leaves.

### 3.6 Ameliorative effect on ulcerative colitis in mice

**Fig. 6:**
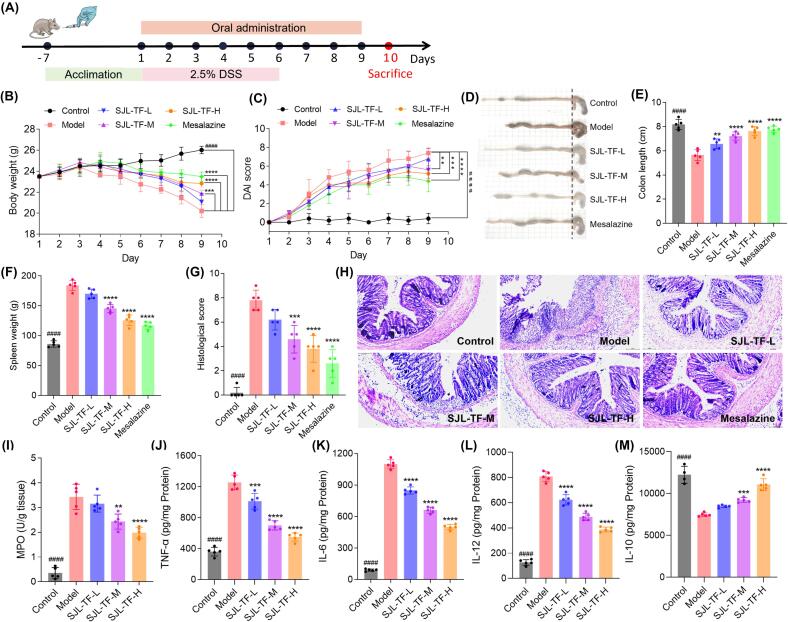
Ameliorative effect of SJL-TF on UC in mice. (A) Experimental design framework; (B) Body weight changes in mice; (C) DAI scores; (D) Representative colon tissue photographs; (E) Histogram of colon length; (F) Histogram of spleen weight; (G) Histological scores based on H&E-stained colon tissues; (H) H&E staining images of colon tissues; (I) MPO activity; (J) TNF-α levels; (K) IL-6 levels; (L) IL-12 levels; (M) IL-10 levels. *p < 0.05, **p < 0.01, ***p < 0.001, ****p < 0.0001 and ^####^*p* < 0.0001 relative to the model group.

According to the treatment protocol depicted in Fig. 6A, the ameliorative effects of SJL-TF on UC in mice were evaluated. As shown in Fig. 6B, the control group consistently gained weight from day 1 to day 9, while the model group experienced significant weight loss. Notably, the administration of SJL-TF-M and SJL-TF-H effectively reduced this weight loss in mice with UC. As shown in Fig. 6C, the DAI scores in each group increased from day 1 to day 9, except in the control group. The model group demonstrated the most significant increase in DAI scores, whereas the treatment groups exhibited a comparatively milder rise. This suggests that SJL-TF may effectively alleviate DSS-induced ulcerative colitis (UC) in mice, particularly in the SJL-TF-M and SJL-TF-H groups. Notably, the DAI scores of the SJL-TF-H group were significantly lower than those of the SJL-TF-M and SJL-TF-L groups starting from day 5. As depicted in Fig. 6D and E, the treatment groups exhibited a significantly longer colon lengths compared to the model group, suggesting that SJL-TF effectively inhibited colon shortening in mice with UC. According to Fig. 6F, the spleen weight in the model group was substantially higher than that in the control group, reflecting pronounced inflammation due to DSS administration. Conversely, SJL-TF treatment led to a marked reduction in spleen weight, reflecting a significant decrease in inflammation. Fig. 6G illustrates the histological scores obtained from H&E staining, which are shown in Fig. 6H. The model group showed marked inflammation in the colonic tissues compared to the control group, characterized by epithelial damage, inflammatory cell infiltration, and a decrease in mucin-secreting goblet cells. Conversely, the SJL-TF treatment groups exhibited improved crypt integrity, indicating that SJL-TF may be effective in alleviating colonic inflammation.

As shown in Fig. 6I, the DSS-induced UC mice displayed an abnormal elevation in colonic MPO levels. In contrast, those treated with SJL-TF-M and SJL-TF-H demonstrated markedly decreased MPO activity, indicating a significant reduction in inflammation. The pro-inflammatory cytokines, including TNF-α, IL-6, and IL-12, were quantified, and the results indicated a significant decrease in their levels following DSS administration, with significant reductions observed after treatment with SJL-TF (Fig. 6J-L). The levels of the anti-inflammatory cytokine IL-10, which reflect the suppression of the inflammatory response, were also measured. It revealed a significant decline in the model group, while a notable increase was observed in the SJL-TF-M and SJL-TF-H groups (Fig. 6M). Therefore, it can be inferred that the flavonoids in sour jujube leaves play a significant role in alleviating DSS-induced UC, particularly by reducing inflammation [36], [37].

## 4 Conclusions

This study optimized the extraction and purification of SJL-TF, demonstrating its potential therapeutic effects on ulcerative colitis. By optimizing the UAE process through RSM, an SJL-TF yield of 48.47 ± 0.36 mg/g was achieved, demonstrating greater extraction efficiency compared to traditional Soxhlet extraction method. The application of polyamide resin chromatography further purified SJL-TF, resulting in an increased purity of 74.58 ± 0.63 %. Additionally, a UPLC-QQQ-MS/MS method was developed and validated, enabling the concurrent detection of ten major flavonoid compounds in the SJL-TF extracts, thereby providing a reliable foundation for quality control. Moreover, the SJL-TF exhibited a significant ameliorative effect on DSS-induced UC in mice by reducing the inflammatory response. Therefore, this study provides valuable insights into the extraction and purification processes, quality control, and beneficial properties of flavonoids from sour jujube leaves, establishing a solid foundation for the future development of innovative treatments utilizing these active compounds.

## CRediT authorship contribution statement

**Hongke Wei:** Writing – original draft, Methodology, Investigation, Data curation. **Xiaomin Wang:** Writing – original draft, Methodology, Investigation, Data curation. **Jiaqi Wang:** Software, Formal analysis, Conceptualization. **Shijie Ren:** Investigation, Data curation. **Luis A.J. Mur:** Formal analysis, Conceptualization. **Dong Lu:** Writing – review & editing, Resources, Project administration, Funding acquisition. **Duo Cao:** Writing – review & editing, Resources, Project administration, Funding acquisition.

## Declaration of competing interest

The authors declare that they have no known competing financial interests or personal relationships that could have appeared to influence the work reported in this paper.
